# Perspectives of migrant men who have sex with men and professionals on personal, social and structural barriers and facilitators to sexual healthcare access and outreach strategies: A qualitative study

**DOI:** 10.1016/j.jmh.2025.100342

**Published:** 2025-06-30

**Authors:** Chrissy PB Moonen, Christian JPA Hoebe, Casper DJ den Heijer, Jill Buursma, Marita ILS Werner, Ymke J Evers, Nicole HTM Dukers-Muijrers

**Affiliations:** a Department of Sexual Health, Infectious Diseases and Environmental Health, Living Lab Public Health Mosa, South Limburg Public Health Service, Heerlen, the Netherlands; b Department of Social Medicine, Care and Public Health Research Institute (CAPHRI), Maastricht University, Maastricht, the Netherlands; c Department of Medical Microbiology, Infectious Diseases and Infection Prevention, Care and Public Health Research Institute (CAPHRI), Maastricht University Medical Center (MUMC+), Maastricht, the Netherlands; d Department of Health Promotion, Care and Public Health Research Institute (CAPHRI), Maastricht University, Maastricht, the Netherlands

**Keywords:** Men who have sex with men, Underserved populations, Health services accessibility, Sexual Healthcare, Health Disparity

## Abstract

**Background:**

Migrant men who have sex with men (mMSM) carry a disproportionate burden of sexually transmitted infections (STIs) yet encounter unique barriers to accessing sexual healthcare. This qualitative study explored mMSM’s and professionals’ perspectives regarding mMSM’s personal, social and structural barriers and facilitators to Dutch sexual healthcare access and outreach strategies.

**Methods:**

A qualitative study was conducted using semi-structured interviews with 15 mMSM (aged ≥16) and 10 professionals from various organisations working with mMSM. mMSM were recruited via sexual health nurses at three STI clinics of a Dutch Public Health Service and flyers; professionals via email, flyers, and the project team’s network. Transcripts were analysed thematically using deductive and inductive coding by two researchers, ensuring intercoder agreement.

**Results:**

Key facilitators for sexual healthcare access included supportive social networks, in-person consultations, and culturally and LGBTQ+-sensitive staff. Main barriers involved fear of stigma rooted in cultural and religious beliefs, limited awareness of available services, and language-related challenges. Suggested outreach strategies included dating app advertisements, short multilingual videos, and translated posters in venues frequented by mMSM (e.g., bars and schools). Notably, most participants accessed services through personal referrals, underlining the importance of including social networks in outreach strategies.

**Conclusions:**

Addressing personal, social and structural barriers while enhancing facilitators contributes to more inclusive and equitable sexual healthcare services. Besides, increasing reach of mMSM through the dissemination of tailored information via social networks, digital platforms, and community settings further supports this objective. These findings inform strategies aimed at reducing health disparities and contribute to broader STI, HIV, and hepatitis prevention goals.

## 1 Background

Each year 2.3 million deaths and 1.2 million cancer cases are caused by sexually transmitted infections (STI), human immunodeficiency virus (HIV) and viral hepatitis (World Health Organization 2022). The Global Health Sector Strategies by the World Health Organisation WHO aim to eliminate STI, HIV, and viral hepatitis as public health threats by 2030, focusing on improving access to care, reducing stigma, and addressing specific vulnerabilities. The strategies provide a framework for enhancing healthcare services, reducing health disparities, and ensuring more equitable healthcare access for key populations.

To achieve these global targets, it is essential to understand the factors that influence access to sexual healthcare, as certain population groups continue to face substantial and persistent barriers (Moonen et al., 2023; Notaro, 2012; Nakakawa et al., 2025). Among these, men who have sex with men (MSM) face heightened risks of exposure to STIs, HIV and viral hepatitis, making their sexual health a priority in many Western countries (Kayaert et al., 2024). Significant differences in the prevalence rates of STIs, HIV, and hepatitis across countries demonstrate that some populations, especially in Sub-Saharan Africa, parts of Asia, Latin America and the Middle East, are disproportionately affected. This highlights the need for low-prevalence countries, like the Netherlands, to intensify efforts to reach migrant MSM (mMSM) for testing (Bhutta et al., 2014; Foundation’s Polaris Observatory, 2023; Fu et al., 2022).

MSM can face several barriers to accessing healthcare, such as sexuality-related stigma (Wao et al., 2016). mMSM may experience additional vulnerabilities, not only related to their sexual orientation but also to their migration background (Bil et al., 2019). These include concerns about their legal status, cultural barriers and discrimination, as well as uncertainty about healthcare rights and language barriers (Egli-Gany et al., 2021; Felipe da Cruz et al., 2023). In addition, research has shown that mMSM are less familiar with preventive measures, such as post-exposure prophylaxis (PEP), pre-exposure prophylaxis (PrEP), and HIV testing compared to non-migrant MSM (Bil et al., 2019; Evers et al., 2023). These inequalities in sexual healthcare access might be, among other things, explained by social and structural determinants, such as economic crises (e.g. cutbacks) and hostile discourse on migration (e.g. anti-immigrant rhetoric) (Egli-Gany et al., 2021). Underutilisation of these preventive health services contributes to bacterial STIs (chlamydia, gonorrhoea, syphilis), HIV, hepatitis B and C virus (HBV, HCV) cases left undiagnosed and untreated within this population.

In several western countries, such as the Netherlands, sexual health care for key/high-risk populations is arranged, consisting of (free) STI/HIV testing, PREP and HBV vaccinations (Evers et al., 2023). Despite increasing recognition of the need for inclusive and culturally sensitive sexual health services, significant knowledge gaps remain regarding mMSM’s and professionals’ perspectives on healthcare accessibility and outreach strategies. Although the active involvement of both target communities and relevant professionals is widely recognised as crucial for ensuring the cultural relevance, accessibility, and effectiveness of such services (McCuistian et al., 2023), empirical studies addressing this knowledge gap remain scarce.

Therefore, this qualitative study aimed to explore personal, social, and structural barriers and facilitators influencing access to and engagement with sexual healthcare services among mMSM in the Netherlands. In addition, we examined perceptions on how to reach more individuals from this key population and whether engaging their social networks could help increase uptake of sexual healthcare.

## 2 Methods

### 2.1 Study design

We conducted a qualitative study to explore the perspectives of mMSM and professionals on personal, social and structural barriers and facilitators to sexual healthcare accessibility, as well as suggested outreach strategies. Semi-structured interviews guided by separate topic lists were conducted with mMSM and professionals who engage with mMSM in their professional roles. For reporting, the COnsolidated criteria for REporting Qualitative research (COREQ) guidelines were followed (Tong et al., 2007) (Appendix 1).

### 2.2 Theory

Thematic analysis was used to structure and analyse the qualitative data (Braun and Clarke, 2006). To ensure a theoretical foundation for the individual level, constructs of the Health Belief Model, including perceived susceptibility, perceived severity, health motivation, perceived facilitators, perceived barriers, and cues to action, were embedded into the topic list to grasp engagement with sexual healthcare (Rosenstock, 1974). Researchers CM and JB integrated all the identified barriers and facilitators into a model based on the Social-Ecological Model, capturing personal to environmental levels: individual, interpersonal, organisational, community, and societal/policy (Bronfenbrenner, 1979).

### 2.3 Context

In the Netherlands, STI testing is provided through general practitioners (GPs), commercial testing services, and Centres for Sexual Health (CSH; i.e. STI clinics), which operate under the umbrella of the Public Health Services (PHS). Even though GPs most often perform HIV and STI tests, the CSH is the sole provider offering free STI testing and counselling to key populations at increased risk of infection, including MSM, sex workers, and individuals under 25 years of age (Bogers et al., 2022). The CSH encourages MSM to undergo annual STI tests, or testing following symptoms, partner notification, or condom failure, and are eligible for free HBV vaccinations (Rijksinstituut voor Volksgezondheid en Milieu (RIVM) 2022).

A sexual health consultation at the CSH can be scheduled via a call centre, after which clients complete an online questionnaire in Dutch or English. During consultation, a healthcare nurse assesses the client’s needs and provides services like STI testing or HBV vaccination. Test samples are sent to a regional hospital for analysis, with negative results communicated via text message. In case of an STI, individuals are phoned and invited to the CSH for follow-up consultation, treatment and partner notification.

### 2.4 Participant selection

Recruitment of mMSM took place via nurses during sexual health consultations at the three STI clinics in the South of Limburg (purposive selection). Participants were selected based on eligibility criteria and demographic characteristics to ensure diversity across multiple migration backgrounds. The inclusion criteria for mMSM were: aged 16 years or older, originating from Eastern Europe, Latin America, Asia, Africa, the Caribbean or Suriname, residing in the region of South Limburg in the Netherlands, assigned male at birth and having sex with other males. Additionally, interviewed mMSM and professionals were handed bilingual recruitment pamphlets to voluntarily distribute within their networks, display in shared places such as asylum seekers centres (ASC), or share at LGBTQ+ events (snowball sampling).

If a mMSM met the inclusion criteria during the consultation, nurses provided a bilingual (Dutch/English) study pamphlet along with verbal information. If the person was interested and gave verbal consent, the contact details were securely passed to the research nurse through the secure CSH system. The research nurse then contacted the participant to schedule an interview. A total of 17 mMSM were recruited, of whom two had to withdraw due to personal circumstances.

Professionals were purposively selected to ensure representation of various roles and organisational perspectives. Professionals were eligible if they had direct professional interactions with mMSM in South Limburg. They were recruited through direct e-mail invitations, via the project group's professional network, and a recruitment pamphlet at a networking event. Ultimately, ten professionals were recruited. Recruitment for both groups took place between December 2023 and April 2024, concluding when data saturation was reached.

### 2.5 Setting

To maximise participant comfort, the interviews were conducted at a location of their choice, including the PHS, participants’ homes, professionals’ workplaces, or online via Zoom when an in-person interview was not feasible. A professional telephone interpreting service was available to bridge language difficulties. The interviews were performed by a female sexual health nurse and a female researcher (CM, MSc). CM has prior experience in qualitative research and is part of a research team with extensive expertise in this field (Unjustified character removed: ]). The nurse led the interviews with mMSM, drawing on her experience in sensitive conversations, trust-building, and engagement with this key population. A trusting relationship had already been established with some mMSM during sexual health consultations with the research nurse. Researcher CM led the interviews with the professionals. We acknowledge that the research team's backgrounds, professional roles, and previous work with migrant and LGBTQ+ communities may have influenced interactions with participants, as well as the collection and interpretation of data.

No individuals other than the nurse and researcher were present during the interviews. At the start of each interview, participants were briefed again on the study's aims, content, and fundamental interviewer characteristics, including age, professional background, expertise, and interest in the topic. After the interview, participants received a goodie bag, while mMSM were additionally reimbursed with a €25 online shopping voucher, condoms, and sexual health information.

### 2.6 Data collection

Two semi-structured topic lists were developed: one for mMSM (Appendix 2) and another for professionals (Appendix 3). Experienced sexual healthcare professionals piloted the lists, and adjustments were made throughout the interview process to enhance clarity and better capture key concepts. After obtaining written informed consent, interviews were audio-recorded and transcribed verbatim by a professional transcription service. Interviews with the mMSM lasted approximately 1.5 h; interviews with the professionals took around 45 min. Participants did not receive transcripts for review, nor were repeat interviews conducted, and no feedback was provided on the findings. We decided to forgo feedback due to safety concerns, particularly as some mMSM had uncertain legal status or had been denied asylum, and could only be reached by phone, making meaningful feedback by all participants difficult.

Topics in the semi-structured interview guide for mMSM included: demographic characteristics, first encounter with the CSH, factors of the Health Belief Model, opinion on Dutch sexual healthcare, strategies for reaching other mMSM, and the role of personal networks and peer referrals in sexual healthcare access.

Topics in the semi-structured interview guide for professionals included demographics, professional role and interactions with mMSM, barriers and facilitators in engaging with mMSM, strategies for increasing the accessibility of sexual healthcare, approaches to reaching mMSM, and social networks (for referral). Also, perspectives on the constructs from the Health Belief Model were explored, e.g. how professionals think mMSM might perceive their susceptibility to STI.

### 2.7 Data analysis

The anonymised transcripts were coded using the qualitative software program ATLAS.ti (version 22), following Braun and Clarke’s thematic analysis framework (Braun and Clarke, 2006). A combination of deductive and inductive coding was applied: initial codes were derived from the topic lists, while emerging themes were identified from the data. Coding was performed by CM (MSc) and second researcher JB (MSc). Discrepancies in coding were resolved through intercoder agreement. After coding, themes were synthesised, and broader categories were established. The codebooks can be found in Appendix 4. To structure the analysis of factors influencing access to sexual healthcare, researchers CM and JB mapped the identified barriers and facilitators onto the levels of the Social-Ecological Model, including the individual, interpersonal, organisational, community, societal and policy levels. To structure the barriers and facilitators across the levels, we differentiated whether each was reported by mMSM, by professionals, or by both groups. Concepts were defined as barriers if their presence impeded accessing or engaging with sexual healthcare and considered facilitators if their presence promoted this.

## 3 Results

### 3.1 Description of the sample

Of the 15 mMSM interviewed, six originated from Central and South America, four from Asia, two from Africa, two from Eastern Europe, and one from the Caribbean (Table 1).

**Table 1:** Characteristics of the mMSM.

Characteristic	N ( %)
Biological gender	
Male	15 (100)
Age (in years)	
18–30	6 (40)
31–40	8 (53)
40+	1 (7)
Country of origin^a^	
Central America	3 (20)
Caribbean	1 (7)
Eastern Asia	1 (7)
Eastern Europe	2 (13)
Northern Africa	1 (7)
South America	3 (20)
Southern Africa	1 (7)
Western Asia	3 (20)
Education level	
Secondary vocational education	3 (20)
Higher professional education	2 (13)
University education	10 (67)
Years in the Netherlands	
0–5	11 (73)
6–10	4 (27)

a Following the United Nations Statistics Division geographic regions (United Nations Statistical Office 2025).

The majority held university degrees, the mean age was 31.7 years (SD = 7.9; range: 20–53), and participants had lived in the Netherlands for an average of 4.1 years (SD = 2.4; range: 9 months-8 years). Nine interviews took place at the CSH, three online via Microsoft Teams, and three at the participants’ residences – including one at an ASC. Ten interviews were conducted in English, four in Dutch and one with the assistance of a Spanish-Dutch telephone interpreter.

Additionally, ten professionals were interviewed, including those working in LGBTQ+ advocacy, migrant support, advocacy organisations, medical care, and politics (Table 2). Their experience in the field varied widely, ranging from 1 to 27 years (*M* = 9.3; SD = 9.3). The interviews with the professionals were all conducted in Dutch. Six took place at the professional's workplace, two at the PHS, one at the professional's home and one at a local university.

**Table 2:** Characteristics of the professionals.

Characteristic	N ( %)
Biological gender	
Male	5 (50)
Female	5 (50)
Organisation	
Organisation for aid to asylum seekers	1 (10)
Civil society organisation	1 (10)
Disease advocacy organisation	1 (10)
General Practice for asylum seekers	2 (20)
General practice	1 (10)
LGBTQ+ organisation	2 (20)
Political Party	1 (10)
Public Health Service	1 (10)
Role	
Chairman	1 (10)
Coordinator	2 (20)
Councillor	1 (10)
Health educator	1 (10)
Nurse	2 (20)
Social cultural worker	1 (10)
LGBTQ+ contact	1 (10)
Sexologist	1 (10)
Years in the field	
<5	5 (50)
5–10	2 (20)
10–15	1 (10)
15+	2 (20)

### 3.2 Barriers and facilitators to sexual healthcare

**Fig. 1:**
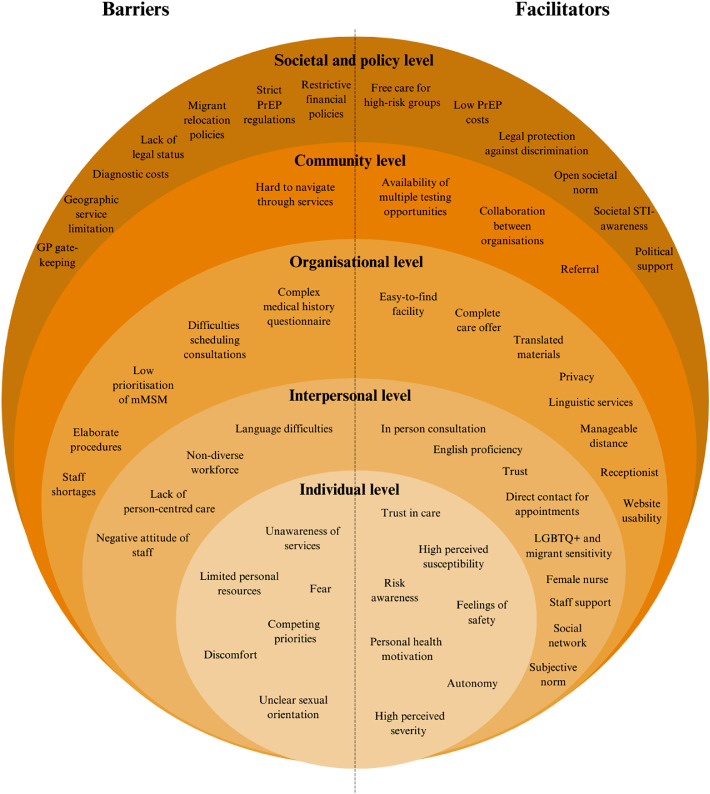
Barriers and facilitators to sexual healthcare access and engagement by mMSM and professionals.

Fig. 1 provides an overview of the identified (perceived) facilitators and barriers mMSM experience in engaging with sexual healthcare, structured within the socio-ecological model. Appendix 5 elaborates on this by presenting the barriers and facilitators, distinguishing those identified by mMSM, by professionals and by both groups. Of note, the barriers in the figure can also be seen as facilitators—and vice versa—if reversed, as each barrier has a corresponding facilitator when framed in an opposite way (e.g., "easy-to-find facility" as a facilitator vs. "hard-to-find facility" as a barrier).

#### 3.2.1 Individual level

##### 3.2.1.1 Perceived susceptibility

Most of the mMSM perceived themselves as highly susceptible to STIs, due to visits to gay saunas and sex parties, and reduced condom use, either due to PrEP use or personal pleasure. Also, the generally higher risk of STI transmission among MSM and sexual encounters with multiple or unknown partners was mentioned. A few mMSM considered themselves at low risk, often due to stable relationships or limited sexual activity. One participant noted a perceived low prevalence in his social circles: *‘’Most people or Asian people from my network, they really care about sexual health. So they are very cautious to protect themselves.’’* (mMSM, Eastern Asia). Other mMSM cited personal STI history (‘’only twice’’), or STI (prevention) awareness gaps as a reason for low perceived susceptibility: *‘’Me, as a young person arriving in the country, I got something (an STI) immediately, because I didn’t think about it. And I wasn’t like properly educated, because it was quite early on when I moved.’’* (mMSM, Southern Africa)*.*

Professionals had varying opinions on the perceived susceptibility to STIs among mMSM. Some generalised that mMSM do not fully recognise their susceptibility due to limited STI awareness. In contrast, one professional suggested that a lack of perceived risk or a sense of reassurance from the availability of healthcare could play a role: *‘’But in every ASC, there’s always one couple who just don't take it seriously — the kind of couple that regularly shows up at the consultation though everything was tested and fine weeks ago, and then they’ve had unprotected sex again.’’* (Professional, GP care for asylum seekers). A few professionals acknowledged their uncertainty, emphasising that perceptions of susceptibility likely differ between individuals.

##### 3.2.1.2 Perceived severity

Having an STIs was often associated with shame, fear, and feelings of impurity among mMSM. The perceived severity was also influenced by whether an infection was curable, with HIV seen as more psychologically and socially burdensome due to stigma: *‘’The first time I was really scared. I had a date with someone and the date, I think a week later or so, he said, ‘I'm positive’. I said, what do you mean, positive? ‘Positive, I have HIV’. And then I was really, really depressed for a few days because I didn't know what to do and you get really scared too.‘’* (mMSM, Western Asia). Some mMSM mentioned that repeated infections led to a decreased perceived severity: *‘’It's very weird to say, but you get used to it with time. If you've had an STI once, the second time it just doesn't get bad (..). You know who to contact, just the first time it's always hard to accept and believe that it will be okay.‘’* (mMSM, Western Asia). One participant highlighted financial and legal uncertainties related to STI diagnoses: *‘’I think it (to get an STI) can be scary. I think it all depends on whether you have insurance here, like wat is your status here in the Netherlands?’’* (mMSM, Eastern Europe). Professional guidance appeared to mitigate these concerns.

Even though most professionals were cautious in generalising statements, one-third of the professionals echoed that mMSM might view STIs in a highly negative light, describing diagnoses as shocking or shameful, affecting both health and social standing: *‘’I think the moment you are indeed labelled HIV-positive in your medical records, you know that things can get a bit trickier in life.’’* (Professional, political party). Some speculated that perceptions of severity varied depending on the individual and the type of infection, with treatable STIs considered less severe. A few professionals also suggested that perceptions of severity might differ across migration backgrounds, possibly influenced by factors such as religion.

##### 3.2.1.3 Personal sexual health(care) motivation

Nearly all mMSM rated the importance of sexual health very highly, with most giving it a 10 out of 10. Key motivations for maintaining their sexual health included avoiding disease and symptoms, safeguarding public health, maintaining intimacy, and not transmitting infections to others: *‘’Well, you know, especially for diseases like HIV that we don’t have any cure for yet, I prefer to stay negative. There are other diseases that are treatable, but still, I want to be responsible, I take care of my own body, so I’m being responsible by not passing it to others, so yes, that is most important. You know, my body is my temple, basically.’’ (*mMSM, South America). Some participants drew comparisons to the poor HIV/STI situations in their countries of origin to emphasise their current high prioritisation of sexual health. The motivation to be sexually healthy was also linked to personal well-being, peace of mind, and a desire to remain generally healthy, although some declared occasional risk-taking: ‘’*I used PrEP. The idea is that I would also use a condom, because of course, that's 100 % (protection). But I have this thing that I still sometimes (…) think about who I can use it (a condom) with or with whom I’m willing to take the risk.’’* (mMSM, Caribbean).

Professionals perceived a generally high motivation among mMSM to engage in sexual health services, including PrEP use, HBV vaccination, and STI testing. They linked this motivation to the desire for safer, more carefree sex, and greater self-awareness. Some professionals had been approached by mMSM seeking specific information, which they saw as a clear indicator of motivation. However, concerns remained about whether mMSM were sufficiently aware of available testing and care options. Religion and individual differences were noted as potential influencing factors, and some professionals remained uncertain about motivation levels and noted that this might be very individual.

##### 3.2.1.4 Other individual barriers and facilitators

Frequently cited barriers by mMSM and professionals included limited awareness of sexual healthcare services as a newcomer in the country, and a fear of stigma or discomfort linked to cultural and religious norms: *‘’Especially talking to doctors about my own body, that was a bit harder. Especially because I come from a whole different culture, where talking about homosexuality is mainly still taboo. So for me, it is a bit embarrassing to talk about.’’* (mMSM, South America). Besides fear of stigma, fear about data confidentiality and a positive test result prevailed. Other issues mentioned by both groups were limited personal resources, including a limited budget and transportation options, and competing priorities during the asylum seekers' process. mMSM also mentioned discomfort with sample collection, distrust in (ASC) care, and being unclear of their sexual orientation. Professionals further noted possible distrust in vaccinations and fear of needles.

Facilitators for mMSM identified by both groups included feeling comfortable and safe during consultations to discuss sexual health-related topics, being personally motivated to know one’s sexual health status, STI risk awareness, and trust in the healthcare system. Professionals also emphasised the perception of autonomy as a facilitator, for example, being able to choose between various sexual healthcare providers and locations, and the course of action concerning testing and treatment.

#### 3.2.2 Interpersonal level

Major barriers at the interpersonal level, identified by both mMSM and professionals, included language difficulties, caused by a language barrier and a lack of multilingual staff. Furthermore, the absence of a culturally diverse workforce was perceived as a limitation in fostering trust and inclusivity. While interpreters can facilitate communication, some professionals expressed concerns about mMSM’s distrust of interpreters, perceiving this as an additional barrier to effective care. Additionally, both groups highlighted negative staff attitudes as a significant obstacle. Instances were reported where healthcare providers exhibited judgmental or dismissive behaviour, such as expressing disapproval of sexual practices or displaying impatience during consultations. mMSM specifically noted a lack of person-centred care at the ASC, describing experiences where their concerns were not taken seriously. Some participants reported a lack of recognition of their sexual orientation, with instances where they felt disbelieved, were never asked about their identity, or perceived a dismissive attitude from healthcare providers.

In-person consultation was stressed as a major facilitator by mMSM and professionals for a positive sexual healthcare experience. Support of staff and network members was mentioned to bridge linguistic differences, navigate care, and schedule appointments. Besides, staff’s LGBTQ+ and migrant sensitivity came up as welcoming: *‘’The CSH as an institution is better than going to the GP, because it is made for this (specialised sexual healthcare) and it is a safe environment where you can feel comfortable and you don’t feel judged by the nurses and all of the people that you are involved with.’’* (mMSM, Southern Africa). Moreover, the staff's positive attitude, characterised by non-judgmental and friendly interactions, was found important. Both mMSM and professionals additionally highlighted the importance of building trust relations and assuring confidentiality: *‘’It’s also a big issue, but it is stated from the start of every consultation that like this (at the CSH) is confidential and we’re not going to share it even within the regular healthcare system, so your data is protected. I think that gives, you know, trust in the organisation and makes it more like, makes you want to go there.‘’* (mMSM, Central America). Consultations with fixed staff also worked in favour of building trust relationships. Besides, direct contact for scheduling appointments, as well as offering services in English, were mentioned as facilitating factors. Notably, some mMSM expressed a preference for consultations with a female nurse, while one professional mentioned a perceived preference for a male staff member.

A few professionals reflected feeling discomfort when discussing LGBTQ+ related topics with individuals from conservative backgrounds. Besides, a single professional identified a lack of internal motivation among colleagues to actively share information, for instance, about LGBTQ+ events or services.

#### 3.2.3 Organisational level

Barriers shared by both groups were hard-to-find facilities and limited privacy in the waiting room area. Besides, an overly elaborate procedure for STI testing and a complex medical history questionnaire with many questions were often viewed as a barrier. Additionally, it was noted that multilingual materials were lacking, even though an English version was appreciated. mMSM additionally reported that they found some questions not to be (culturally) translatable for newcomers. For example, Dutch education levels are sometimes not directly transferable to other countries. Besides, some mMSM found the questions confrontational and overly direct, which they were not accustomed to*: ‘’But at the beginning, yes, I was eh, I don’t know, shocked for a little bit, like these are very, very intimate questions.‘’* (mMSM, South America).

Additional barriers were difficulties in scheduling appointments, including long waiting times for consultations and limited availability of contact hours. Other obstacles included inconvenient early morning consultation slots, the lack of walk-in hours, and restrictions on scheduling appointments on behalf of others. Moreover, an incomplete sexual healthcare offer—such as the absence of anal swabs at non-SHC clinic sites—was mentioned by both professionals and mMSM. mMSM also reported that a lack of clear information regarding free healthcare services, policies on travel expense reimbursement, and the confidentiality of services discouraged engagement. Professionals similarly highlighted insufficient consultation time and the complexity of procedures for STI testing as key challenges. Additionally, singularly, staff shortages and the systemic under-prioritisation of (m)MSM within sexual healthcare services were cited as barriers to improving accessibility.

Both groups valued a manageable distance to the care facility, privacy in waiting and consultation rooms, comfortable facilities, clear and comprehensive information during consultation, and the availability of linguistic services, such as interpreter availability. mMSM also highlighted good accessibility by public transport and the ease of scheduling appointments: ‘*’I mean just to be able to get an appointment was really easy, right, I think you just need to know about it.’’* (mMSM South America). A receptionist at the entrance of a building or office could help in the findability of the facility. mMSM also valued a user-friendly website, including accessible contact details, an online booking system, and urgent appointment options, while professionals emphasised fixed service locations.

#### 3.2.4 Community level

Barriers at the community level were minimal. Both groups acknowledged that it can be hard to navigate through the (sexual) healthcare landscape as a newcomer:*‘’(..) At the beginning I got confused a lot, because I didn’t know the names of the stuff in here. I don’t know the ‘GGD’ (PHS), what does it stands for (..).‘’* (mMSM, Central America). One professional noted a failure to refer mMSM for free HBV vaccinations, as this was not sufficiently embedded in their protocols. Facilitators were similarly few but included sufficient availability of multiple STI testing opportunities, including home testing and multiple CSH locations in one region. Professionals saw inter-organisational referral between (healthcare) organisations and collaboration as beneficial.

#### 3.2.5 Policy and societal level

Both mMSM and professionals identified significant policy-level barriers that limit access to sexual healthcare. A major concern was the restrictive financial policies affecting budgets for STI testing and outreach initiatives. Additionally, several policy frameworks were seen as obstructive, including strict and frequently changing PrEP regulations, national migrant relocation policies that disrupt continuity of care, and the lack of legal status, which prevents reimbursement for healthcare services. Moreover, some mMSM highlighted the high diagnostic costs associated with GP care and the gatekeeping role of GPs in accessing specialist care as key obstacles. For the CSH, there is a geographic service limitation- individuals are only permitted to access services within their designated region. Some mMSM mentioned this as a barrier, as they were uncertain about which CSH they were allowed to visit.

Facilitating factors included the provision of free sexual health care for high-risk groups and the accessibility of PrEP at reduced costs. Furthermore, some mMSM perceived a high level of societal awareness regarding STIs and societal norms that encourage openness about sexual health: *‘’Like the conversations are usually really open. It is also more of a sex-positive country, I would say, than my country, which is much more conservative.’’* (mMSM, South America). Additionally, one mMSM highlighted feeling safe by legal protections against discrimination, as discrimination is legally banned in the Dutch constitution. Furthermore, some professionals underscored the role of political support in enabling access to sexual health care, particularly through its influence on funding availability.

### 3.3 Cues to action and increasing outreach

#### 3.3.1 Social network for referral

Both groups emphasised that support from one's social network played a crucial role in access to sexual health services. Social networks were seen as a crucial conduit for information exchange and referrals: *‘’The people who have experienced that it is reliable, that is your word of mouth. They actually directly ensure that next year there will be another group sitting in front of you.’’* (Professional, patient advocacy organisation). mMSM often became aware of the CSH through peers—friends, roommates, or sex partners—and nearly all had already adopted an informal peer referral role: *‘’It depends, I mean it depends on like how close eh, we are, but yes, I just talked to a migrant also from Slovakia a week ago and he is also getting PrEP eh, and going regularly (to the STI clinic). So I am someone who will bring up this topic (…). When I feel like it's relevant on the table, I will always bring it up.’’* (mMSM, Eastern Europe). Most mMSM had other MSM in their social network and expressed comfort in discussing sexual health topics within their trusted circles. When asked about conversations regarding STI testing and sexual healthcare, the openness within their network was often emphasised: *"Sure, (I talk about) everything. I even force it sometimes."* (mMSM, Western Asia). Professionals largely supported this finding, noting that close social networks foster a sense of safety and trust. However, fear due to cultural taboos or concerns about disclosure, particularly within some migrant communities, for example, in ASCs, was noted as a key challenge that could prevent individuals from seeking or recommending care.

#### 3.3.2 Preferred communication channels and outreach settings

Both mMSM and professionals emphasised the importance of combining digital outreach with physical, community-based approaches to reach mMSM populations effectively. mMSM participants consistently recommended social media, particularly dating apps such as Grindr and Tinder, as the most effective and discreet tools for disseminating information about sexual healthcare. Similarly, professionals acknowledged social media as a useful method, although they prioritised outreach within ASCs, through queer organizations, and via municipal communication channels. To improve digital outreach, mMSM participants proposed enhancing the accessibility and visibility of sexual healthcare service websites. Suggestions included incorporating short, multilingual video clips that explain available services and provide clear navigation guidance, particularly for non-native speakers or newly arrived migrants.

Physical outreach strategies included posters and flyers placed in hospitality venues (e.g., bars, clubs, saunas), school restrooms, campus intranets, municipal buildings, ASCs or onboarding kits for new international students. Both mMSM and professionals also recommended workshops or testing days at ASCs to raise familiarity with sexual health services. Several additional outreach ideas were suggested by individual respondents, including the deployment of a mobile testing unit, information dissemination on public buses, targeted outreach in companies employing (seasonal) migrant labourers, and forming partnerships with local community groups to facilitate group discussions in safe settings. Professionals shared similar views regarding the importance of visual materials but placed greater emphasis on involving local stakeholders or partnerships with queer organisations. Approximately one-third of the professionals indicated that appointing an LGBTQ+ ambassador could enhance outreach to mMSM by lowering thresholds for engagement. Professionals also suggested integrating sexual health testing into municipal structures to strengthen local service provision.

Both participant groups supported the use of a neutral referral tool, such as a multilingual business card containing key information about the CSH.

The majority of the mMSM reported a willingness to distribute such a card, motivated by a perceived importance of sexual health. Still, several barriers were noted, including the perception of sexual health as a private matter, language barriers, and uncertainty regarding others’ sexual orientation. Conversely, facilitators mentioned by mMSM included a trusting relationship, empathy with the LGBTQ+ community, and previous positive experiences with the CSH. Professionals additionally highlighted that low literacy and lack of access to smartphones could hinder the effective use of the card, especially with features such as QR code scanning. Some professionals also raised concerns about potential misuse, noting that individuals outside the intended target group might take undue advantage of the free services. Nonetheless, all professionals expressed a willingness to distribute the referral card within their professional context to more effectively reach mMSM for sexual healthcare services.

#### 3.3.3 Key messages for outreach

mMSM and professionals agreed that outreach messaging should primarily raise awareness and clearly communicate the free services offered by the CSH, including STI testing, HBV vaccination, consultations, and confidentiality guarantees. Both mMSM and professionals emphasized the importance of clear contact details, links to the website, and booking information. Both groups underscored the importance of concise, plain language, translation into multiple languages, and visually appealing materials. Other, less frequently mentioned suggestions included highlighting symptoms and risks, the importance of knowing one’s sexual health status, the availability of interpreters, and reimbursement of travel costs for individuals in asylum seekers’ centres. One mMSM stressed the need to reduce stigma by normalising sexual health discourse and promoting positive messaging around sexuality and pleasure. Some professionals noted that it is important to highlight the well-organised nature of healthcare in the Netherlands; for example, (negative) test results are communicated efficiently via text messages by the CSH.

## 4 Discussion

The findings of this study provide insights into the sexual healthcare accessibility for mMSM in the Netherlands and outreach strategies by exploring mMSM’s perspectives and perspectives of professionals on individual, social and structural barriers and facilitators. Most participants perceived a high susceptibility to STIs, which related to risk behaviours and increased STI rates among MSM. Severity was shaped by stigma and emotional burden, particularly for HIV, though repeated STI-infections sometimes reduced concern. Motivation for sexual health(care) was generally high. However, awareness of available services was limited, particularly during the initial period after migration, when individuals were still unfamiliar with the country's (sexual) healthcare system. Previous studies among migrant populations have similarly identified limited awareness of available services as a key barrier to accessing sexual health care (Aibangbee et al., 2024; Baroudi et al., 2020; Parra et al., 2024).

Perceived barriers and facilitators spanned individual (e.g. fear of being stigmatised, trust in care), interpersonal (e.g. language difficulties, LGBTQ+ and migrant sensitivity of staff), organisational (e.g. complex medical history questionnaire, easy-to-find-facility), community (hard-to-navigate services, availability of multiple testing opportunities), and policy/societal levels (e.g. restrictive financial policies, open societal norm). Finally, peer networks for discussing sexual health(care) and guidance toward services were crucial cues to action. Similar influencing factors were found in two reviews among migrant populations, highlighting key barriers to HIV testing and sexual healthcare access, including stigma, cultural and religious norms, migration status, and limited health system accessibility (Felipe da Cruz et al., 2023; Parra et al., 2024).

Our findings show that barriers and facilitators to sexual health services for mMSM emerge at multiple levels. These include personal, social, and structural factors that often overlap, reflecting what has been described in the literature as 'intersectional stigma’ (Turan et al., 2019). Migration status, sexual orientation, ethnicity, and socioeconomic conditions do not operate in isolation but intersect, shaping both access to care and experiences within healthcare systems. As highlighted by others, failing to account for these overlapping forms of stigma and disadvantage can limit the effectiveness of health interventions (Bowleg, 2012; Turan et al., 2025). Future research and practice should therefore adopt an intersectional approach to better understand the complex realities faced by mMSM.

To lift the identified barriers and promote the facilitators of accessing sexual healthcare, we further highlight three practical recommendations. First, navigating unfamiliar healthcare systems and accessing relevant information on services can be challenging. This issue is also reported in recent Dutch studies among heterosexual and Sub-Saharan African migrants (Martinez et al., 2024; Shobowale et al., 2025). Taking this into account, and considering that the main route to the CSH is through peer referral, we recommend exploring social network-based approaches (Choong et al., 2024) and enhancing the accessibility and visibility of information on sexual healthcare services. This can include well-structured, user-friendly websites translated to multiple languages, with clear contact details and addresses.

Second, the testing and the associated information should be simplified, more user-centred and stigma free. Testing could be made easier by reducing the steps to a test by, for example, introducing walk-in hours or an online system for booking an appointment (Martinez et al., 2024). Importantly, pre-test materials—such as medical history questionnaires and brochures—should be short, understandable (B1 language level), and translated (culturally) appropriately. Emphasising confidentiality and offering support for travel expenses may reduce shame, fear of stigma, and financial concerns. While in-person consultations were highly valued by care-engaged mMSM, home-based testing may improve uptake among more care-avoidant individuals by taking away barriers such as shame or travel expenses. Although available for MSM in our region, such testing requires adaptation for mMSM and other key populations (Goense et al., 2025).

Third, we would recommend strengthening staff’s competence in interacting with this key population, especially regarding LGBTQ+ and migrant sensitivity. The value of culturally sensitive care was underscored by a needs and assets assessment conducted in the Euregion Meuse-Rhine (Nakakawa et al., 2025). Beyond cultural sensitivity, participants valued professionalism, non-judgmental and positive attitudes. The competence of healthcare workers has been shown to significantly contribute to the success of HIV/AIDS control programs (Byamugisha et al., 2024). Training staff on cultural backgrounds, inclusive language, and the specific needs of migrant MSM could improve trust, comfort, and ultimately, engagement with care services.

### 4.1 Strengths and limitations

Some limitations of this research need to be acknowledged. The primary recruitment of mMSM took place during consultations at the CSH, resulting in a sample that was already engaged with sexual healthcare. Consequently, participants were more likely to consider sexual health important and to possess the awareness needed to access relevant services. However, their experiences with sexual healthcare were essential to understanding both the barriers and facilitators they perceived. Additionally, individuals with a higher level of education were overrepresented in our sample. Despite these considerations, we included mMSM from a diverse range of countries, capturing a variety of cultural backgrounds and experiences. The involvement of professionals from diverse roles and organisations offered a multifaceted perspective on sexual healthcare accessibility.

## 5 Conclusion

Enhancing the accessibility of sexual health services for mMSM requires a multifaceted approach that addresses barriers and reinforces facilitators on multiple levels while considering intersectional stigma. Suggested outreach strategies were dissemination of culturally tailored information via social networks, digital (social media) platforms, and community-based settings. These approaches hold potential to foster more inclusive and equitable sexual health systems, accelerating progress toward the global goal of eliminating STIs, HIV, and viral hepatitis as public health threats by 2030.

## Declarations

### Funding

This work was supported by the National Institute for Public Health and the Environment [no funding number]. The funder had no role in the study design, in the collection, analysis and interpretation of data, in the writing of the report, or in the decision to submit the article for publication.

### Ethics statement

The study, including the protocol, participant information form and written informed consent form, was approved by the medical ethical committee of the Faculty of Health Medicine and Life Sciences of Maastricht University (FHML-REC) (reference number FHML-REC/2023/096).

### Availability of data and materials

The data of this study contain potentially identifying and sensitive participant information. Due to the General Data Protection Regulation, it is not allowed to distribute or share any personal data that can be traced back (direct or indirect) to an individual. In addition, publicly sharing the data would not be in accordance with participants’ consent obtained for this study. Therefore, data used and/or analysed during the study are available from the head of the data-archiving of the Public Health Service South Limburg on reasonable request. Interested researchers should contact the head of the data-archiving of the Public Health Service South Limburg (Tamara Kleine: tamara.kleine@ggdzl.nl) when they would like to re-use data.

## Declaration of generative AI and AI-assisted technologies in the writing process

During the preparation of this work, the authors used ChatGPT (OpenAI Inc) version 3.5 to improve the readability and language of the manuscript. After using this tool, the authors reviewed and edited the content as needed and take full responsibility for the content of the published article.

## CRediT authorship contribution statement

**Chrissy PB Moonen:** Writing – original draft, Visualization, Project administration, Investigation, Funding acquisition, Formal analysis, Conceptualization. **Christian JPA Hoebe:** Writing – review & editing, Supervision, Funding acquisition, Conceptualization. **Casper DJ den Heijer:** Writing – review & editing, Supervision, Project administration, Funding acquisition, Conceptualization. **Jill Buursma:** Writing – review & editing, Visualization, Formal analysis. **Marita ILS Werner:** Writing – review & editing, Project administration, Conceptualization. **Ymke J Evers:** Writing – review & editing. **Nicole HTM Dukers-Muijrers:** Writing – review & editing, Supervision, Funding acquisition, Conceptualization.

## Declaration of competing interest

The authors declare that they have no known competing interests that could have appeared to influence the work reported in this paper.

## Appendix Supplementary materials

Image, application 1

Image, application 2

Image, application 3

Image, application 4

Image, application 5
