# Supplementary material for: Perspectives of migrant men who have sex with men and professionals on personal, social and structural barriers and facilitators to sexual healthcare access and outreach strategies: A qualitative study
Source: J Migr Health. 2025 Jun 30;12:100342. doi: 10.1016/j.jmh.2025.100342 (PMC12273491; doi:10.1016/j.jmh.2025.100342)
Supplement: Supplementary file 2 [file mmc2.docx]

**A.2 Topic list mMSM**

**Table A.2: Semi-structured topic list for interviews with mMSM**

| Topic | Subtopic | Question |
| --- | --- | --- |
| Procedure | Participant information | Have you read the participant information? Any questions regarding this?  This is a confidential conversation. You may stop at any time. The interview will be recorded and written out anonymously. This means that the interview cannot be traced back to you. |
|  | Informed Consent form | Signed?  Do you have any questions before we start?  **Action: Start recording.** |
| Demographic characteristics | Intro | *Let's get to know each other a little better first. I am (...).* |
|  | Introduction | Could you perhaps introduce yourself? |
|  | Age | How old are you? |
|  | Origin | Where were you born? Did you grow up there too? |
|  | Years in the Netherlands | How long have you been in the Netherlands? |
|  | Education level | Did you go to school there/here? What education did you follow?  In case of foreign study: do you know which study in the Netherlands this is comparable to? |
| Route to the PHS/CSH | First encounter PHS | How did you find/come to the Centre for Sexual Health? How was that for you (easy/difficult)? |
|  | Ease of first encounter | How was that for you (easy/difficult)? |
|  | Familiarity with PHS | Were you already familiar with the Public Health Service and the Centre for Sexual Health? |
| Sexual health | *Intro* | *We are now going to talk about sexual health. You do not have to tell us what your sexual health is like at the moment, but we are interested in what you understand by sexual health and whether it is important to you.* |
|  | Understanding of sexual health | What do you understand as sexual health? |
|  | Rating importance sexual health | On a scale of 1-10, how important is sexual health to you right now? (1 is not important at all and 10 is super important). |
|  | Explanation of rating | What makes you give the rating ...? |
|  | Needs for sexual healthy life | What do you need to live a sexually healthy life? |
| Vision sexual healthcare | *Intro* | *We are now going to talk about the positive and negative things you experience when you access sexual health care. You can think of things to do with yourself, your community and the healthcare system.* |
|  | Vision Dutch sexual healthcare | What do you think about sexual health care in the Netherlands? General? GP? PHS? |
|  | Barriers healthcare access | What makes it difficult(er) for you to get sexual health care? |
|  | Facilitators healthcare access | What makes it easy(er) for you to get sexual health care? |
|  | Preferred way of access | In what ways would you like to access care? (This could include going to a location, but also, for example, home tests or online consultations). |
|  | Difference test/vaccination locations | For you, is there any difference how or where you would prefer to be vaccinated and tested? |
| Reach of migrant MSM | *Intro* | *We talked about your own vision. We want to reach the widest possible audience for sexual health care. We are curious about how you think we can reach others MSM migrants. Could we ask a few questions about that?* |
|  | Own vision of reaching MSM | What or where do you think is the best way to reach other MSM born abroad for sexual health care such as STI testing and vaccinations? |
|  | Message for outreach | Which message do you think would be appropriate? |
| Network | *Intro* | *We would like to get a better idea of what your social and sexual network looks like. May we ask you some questions about this?* |
|  | Steady sex partner | Do you have a steady sexual partner? |
|  | Casual sex partners | In the past 6 months, have you had any casual/loose sex partners? |
|  | Origin sex partners | Which countries are your sex partners from? |
|  | Migrant MSM in social network | Are there any other men who have sex with men not born in the Netherlands that you hang out with? (family, friends, acquaintances) |
|  | Discussing sexual health with network | Do you ever talk to them about sexual health care? Or with anyone else? |
|  | Subjective norm sexual healthcare | What do they think about sexual health care? |
|  | Peerfunction for referral sexual healthcare | Would you also advise other migrant MSM to go to the CSH for sexual health care, such as testing for STIs or hepatitis b vaccination? If yes, how? If no, why? |
|  | Business card opinion | What do you think of this card? Content-wise and visually? |
|  | Willingness to use business card | Would you give them this card (action: show card) with information about the CSH? Why (not)? |
|  | Barriers referral PHS | What makes it difficult(er) to refer other migrant MSM to the CSH? Again, you can think of factors affecting yourself, your community or the healthcare system. |
|  | Facilitators referral PHS | What makes it easy(er) to refer other migrants to the CSH? |
| Own input | Further additions | Is there anything you would like to add to the interview or ask us? |
|  | Experience interview | How did you experience the interview? |
|  | Thank you | Thank you for your time. |
