# Supplementary material for: Perspectives of migrant men who have sex with men and professionals on personal, social and structural barriers and facilitators to sexual healthcare access and outreach strategies: A qualitative study
Source: J Migr Health. 2025 Jun 30;12:100342. doi: 10.1016/j.jmh.2025.100342 (PMC12273491; doi:10.1016/j.jmh.2025.100342)
Supplement: Supplementary file 3 [file mmc3.docx]

**A.3 Topic list professionals**

**Table A.3: Semi-structured topic lists for professionals**

| Topic | Subtopic | Question |
| --- | --- | --- |
| Procedure | Participant information | Have you read the participant information? Any questions regarding this?  This is a confidential conversation. You may stop at any time. The interview will be recorded and written out anonymously. This means that the interview cannot be traced back to you. |
|  | Informed Consent | Signed?  Do you have any questions before we start?  **Action: Start recording.** |
| Introductions | Introductions | *Let's get to know each other a little better first. I am (...). Can you also introduce yourself?* |
| Demographics | Age | How old are you? |
| Workplace | Organisation | Which organisation and department do you work at? Can you tell a bit more about what your organisation/department does? |
|  | Role | What is your role within this organisation? |
|  | Field experience | How long have you been working in this field? |
| Organisation | STI role organisation | What do you/your organisation do in terms of STI (prevention or curation)? |
|  | STI policy | Is there an STI (hepatitis B, hepatitis C and HIV) policy in your department/organisation? (if applicable) |
| Contact with mMSM | Way of contact | How do you come into contact with migrant MSM during your work? |
|  | Frequency contact | How many times a week do you come into contact with migrant MSM? |
|  | Perceived reach | Do you feel you are reaching migrant MSM? |
|  | Facilitators reach | What helps (or facilitates) with this reach? |
|  | Barriers reach | What does not help with this reach? |
| Health belief model | Presumed susceptibility STI | How susceptible do you think migrant MSM feel to STIs? |
|  | Presumed severity STI | How do you think migrant MSM view STIs and getting STIs? |
|  | Presumed sexual healthcare motivation | How motivated do you think migrant MSM are to use sexual health services (STI testing, hepatitis B vaccinations, PrEP)? |
|  | Presumed facilitators sexual healthcare | Do you think they see facilitators or benefits of pre-use of sexual health services, such as STI and HIV testing and vaccination? If yes, which ones? If no, why not? |
|  | Presumed barriers sexual healthcare | Do you think they see barriers to using sexual health services? If yes, which ones? If no, why not? |
| Reach mMSM | Best way to reach mMSM | How do you think we can best reach the migrant MSM community for sexual health care? |
|  | Message outreach | What message do you think would be appropriate to reach migrant MSM for sexual health care? |
|  | Increasing sexual healthcare access | How do you think we can make sexual healthcare more accessible to migrant MSM? |
|  | Social network for reaching mMSM | Do you think the social network of migrant MSM can be used for outreach to this group? If not, why not? If yes, how do you think the social network can be used? |
|  | Barriers to using social network | What impeding factors do you expect in engaging the social network for reaching migrant MSM for sexual health services? |
|  | Facilitators for using social network | What facilitating factors do you expect in this regard? |
| Business card | Professional opinion business card | (Show business card). What do you think of the following card? |
|  | Expectation spread network | Do you think this would be spread within the network of migrant MSM? |
| Familiarity with CSH | Familiarity PHS services | To what extent are you familiar with the work of the Centre for Sexual Health? (For people at risk/MSM: testing, vaccination, education/outreach, sense, limburg4zero/this-tests. For professionals: advice, and support. Research). |
|  | Collaboration PHS | Are you already working with the CSG? How is this collaboration going? How can we further improve our cooperation? |
| Wrap up | Additions | Is there anything else you would like to add or ask yourself? |
|  | Experience | How did you experience the interview? |
| Action | Stop recording | Thank you for your participation. |
