# Supplementary material for: Perspectives of migrant men who have sex with men and professionals on personal, social and structural barriers and facilitators to sexual healthcare access and outreach strategies: A qualitative study
Source: J Migr Health. 2025 Jun 30;12:100342. doi: 10.1016/j.jmh.2025.100342 (PMC12273491; doi:10.1016/j.jmh.2025.100342)
Supplement: Supplementary file 4 [file mmc4.docx]

**A.4 Codebooks
Table A.4.1: Codebook migrant mMSM**

| Group | Code | Description | Example from data |
| --- | --- | --- | --- |
| Demographic characteristics | **Age** | The age of the participant in years | “I am 23 at the moment, but soon to be 24.” |
|  | **Origin** | The participant’s country of birth | “Yes, I am (name), I am from Jordan.” |
|  | **Years in the Netherlands** | The duration the participant has been in the Netherlands in years | “Yeah, a year and a half, yeah.” |
|  | **Education level** | The education level of the participant | “Eh, I did my bachelor and eh, master in China. I did a double degree master in Italy.” |
|  | **Living situation** | Current living situation of the participant | “Um, I'm alone, so um, so um- I live in Maastricht by the way.” |
|  | **Status** | Current migration status of the participant | “I mean, it’s the whole procedure. I still  live in here in the azc, so basically it’s there and  then the next part of the process is find a place and  be relocated nearby, I mean, here in Maastricht or  in the city.” |
|  | **Working situation** | Current working situation of the participant | “Well, so it's hard, like job or- Now I am not  working even though I seeked employment in the  past.” |
| HBM / barriers and facilitators to sexual healthcare | **Ease of first encounter** | The level of ease or difficulty of the first encounter with (finding) the PHS | “Yes, it was hard to find because they give you the address, but I didn't know I had to walk like, from the entrance to this office. Like, I was clueless at the time. So I got- I was like, you know, searching with the phone, trying to figure it out and I just walked. And even when I came here to the office, I was like, where is the GGD and the reception? They told me it's like, by the end of the hallway. And then I saw the little banner they had and go in.” |
|  | **Familiarity with PHS** | Familiarity with the Public Health Service before first encounter | “No, not until I came the first time. I asked and I heard about all the stuff that you do here, but-” |
|  | **Understanding of sexual health** | What the participant understands as sexual health | “It is a to be responsible eh, and aware that  you are in danger when you do not protect. Sexual  health it is a eh, knowing that you are in risk when  you are not using protection and act on it like eh, go  to the huisarts, or to the GGD and get advice, stay  negative in all these diseases. So that is sexual  health, like being responsible with me, but also with  the other I have sex with. So yes, and stay healthy.  That, that is what I think it is.” |
|  | **Rating importance sexual health** | The importance of sexual health to the participant on a scale of 1-10 (1 is not important at all and 10 is super important). | “It is very, it is very important, ten.” |
|  | **Explanation of rating** | Explanation of the rating of the importance of sexual health for the participant | “Well, you know, especially like diseases like HIV  that we don’t have any cure anymore. Or not yet, so  I, I prefer to stay negative there the other diseases  are treatable, but still, I want to be responsible, I  take care of my own body, so and I’m being  responsible by not eh, passing it to others, so yes,  that is most important. You know, my body is my  temple, basically.” |
|  | **Needs for sexual healthy life** | The participant’s needs for a sexually healthy life | “Eh, okay so eh, when I check the dating app, it is better I can know some information and also I try to protect myself, always use condom, if not, I would take the PrEP, but it is not, you know, PrEP is not for a general STD, but yes. X2: And what do you mean by on a dating app? What, what eh? Y: So I, so some people, they will eh, write in their profile whether they are taking PrEP or whether they tested before like when they did a, a study test. X2: Test. Yes, and that is important for you to know eh, to and, and so if you know that, then the chance to, to get something is less. Is that what you mean? Y: Yes, yes, yes, yes, yes. X2: Okay. And you said condoms and, and PrEP eh, that will help to, to stay healthy, healthy sexual then. Y: It is not 100%, but at least, it will give some protection, yes. X2: Protection, yes. X1: And how do you know this eh, information on the profile. How do you know it is true? Or is it trust that you build with somebody? Y: Eh, yes, so at the very beginning, if you just look at the profile, it is not very eh, I, I, I don’t know whether it is trustworthy or not, but you know, we will have conversation or something and we will talk about it, you use condom or you have only safe sex. Yes.” |
|  | **Barriers healthcare** | Experienced barriers to access or getting sexual healthcare | “And um, some people are afraid to come up against someone.” |
|  | **Facilitators healthcare** | Experienced facilitators to access or getting sexual healthcare | “Yes, I think it is great to just have the access,  given by the government, right, it is completely  different in my country. You just don’t have that. So  I think it is amazing that you have a place where you  can test for free, where you can just go and feel safe  about it, like the conversations are usually really  open. It is also more of a sex like positive country, I  would say, than my country, which is much more  conservative. So I feel like it is great, it is so like first  world. You can go and have these kinds of conversations and to be able to be tested. I was not on PrEP since the beginning obviously alright, so to just be able to receive the information there and  get the booklet and being asked like constantly as well hey did you think about it, I think it would be great for you, you know. So I think it is good, I really do. I am really happy with it.” |
|  | **Preferred way of access** | Preferred way to access sexual healthcare (going to the PHS, online, etc.) | “I like to do it face to face, yes. I think it is  important, you have to go to do a checkup anyway,  right, so I think it is important to just do it face to  face. I would not mind if I could just do the  appointments online obviously, right, that is faster  and I can safe some time. Also at the beginning, like  the first time you make the appointment, it is  always a bit like kind of nervous, so it is a bit more,  like you can make it a bit more like anonymous in  the way of just doing it online, it always helps, I  think. Right. Especially the people that are more  afraid of talking about it, you know?” |
|  | **Difference test/vaccination locations** | Preferred ways to access care for vaccinations and testing | “I mean, if it is vaccinations related to sexual like..  or stuff like that, then I am happy to do that  through the GGD as well, because that is my go-to  source for sexual stuff, you know. The same is for  my friends. Usually you go through your GP, you  know you just, it is like too expensive. Every time  you want to get like a test and stuff like that, so I  would just rather, if it is a benefit that comes from  the GGD, I am more than happy to take it, yes.” |
|  | **Opinion on Dutch sexual healthcare** | Opinion on the sexual health care in the Netherlands | “The topic of sexual health is something that in  my culture is taboo, um, there can be very- There can be  not talk about experiences and so on, how you feel about that. Um, that way not distributed the right information about that and it has to very often happen in secret. Um, here that's  all more normal, um, so it's talked about at school,  already talked about, also between adults, which that  makes that a lot of problems can be prevented that way because there is a lot more  information and it um, doesn't all have to be done in secret  has to be.” |
| Cues to action and increasing outreach | **First encounter PHS** | How the participant has first found/came to the Centre for Sexual Health (CSH) | “I started meeting people while I was in Maastricht, and they told me about the GGD. But I was just like, whenever someone tells you something exists, but you don't know where to pin it down and how to reach them. So I was just like clueless, like I know it existed, but I thought maybe they're all- I thought actually it was an NGO and I am not sure if it is an NGO, but it seems like more, very structured organization like, from the umbrella, the health care system of the country. So I thought, maybe I can just find another one, like whatever. But then through a chat group for asylum seekers someone did recommend the GGD, and they were like, you can get tested here and you can make an appointment. And I did, I called in and make an appointment, which- Sometimes it's hard because you don't know, they transfer you, you don't call the right number and- Well, finally I got the appointment, and it was here in Maastricht. And I came with, I am not sure, a while back, but I think so, a girl with the name (…) who works here.” |
|  | **Steady sex partner** | Whether the participant has a steady sex partner | “Um, not at first. But now it's just more solid.” |
|  | **Casual sex partners** | Whether the participant has had any casual/loose sex partners in the past 6 months | “No, I don't have a steady one at the moment.” |
|  | **Origin sex partners** | The origin of the sex partner(s) | “Yes, um, what I said, just um, um, I did have an um, Moroccan friend. And from Syria. And um, that was, two Syria and then a Moroccan, which I guess I just, um, most.” |
|  | **mMSM in social network** | Whether there are any other men who have sex with men not born in the Netherlands in the participant’s network | “Sure, I have um, that, that, that young man who has been with me four years ago, who is my best friend right now. Other friends who live in um, Holland.” |
|  | **Discussing sexual health with network** | Whether the participant talks to other migrant MSM in their network about sexual health(care) | “Yeah, I sometimes see someone sitting across from me and I- For me, it's well known that you like the other person, you don't talk about it if it doesn't sound because of that I'm not open either, so um, so I'm not going to um, talk to everybody about it. But I, I- If it's something very clear and he asks a question, then, then I give everything I um, what I know.” |
|  | **Subjective norm sexual healthcare** | The subjective norm of the migrant MSM in the network of the participant regarding sexual healthcare | “Very positive. Just attention given and just explanation.” |
|  | **Peerfunction for referral sexual healthcare** | Whether the participant would advice other migrant MSM to go to the CSH for sexual health care, such as testing for STIs or hepatitis b vaccination and why | “It depends, I mean it depends on like how close eh, we are, but yes, I just talked to ehm, a such migrant also from Slovakia a week ago and he is also getting PrEP eh, and going regularly. So, so I am someone who will bring up this topic, I do not have ehm- When I feel like it's relevant on the table, I will always bring it up. Eh, but I don't know if it's like a common topic eh, people discuss. X1: But if, if it comes up, you talk about it. And then do you also advise to, to go for a test or, or vaccination? Y: Yes.” |
|  | **Own vision of reaching MSM** | Opinion on the place / method to best reach other MSM born abroad for sexual health care such as STI testing and vaccinations | “But I think it’s because it’s not widely communicated. I think also the COA posting like, a flyer, I don’t think I’ve ever seen that it my azc or any temporary camp. X2: There is no flyer from the- Y: Or like, a poster saying, need sexual health, come to us, we’re like, super discreet, you can test here.” |
|  | **Message for outreach** | Opinion on which message would be appropriate to reach other migrant MSM for sexual healthcare | “Yeah. And I think on, I think you might be able to paste links in, in the bio on Grindr, so that may also- Like, just link to the website, but I’m not, I’m not really sure.” |
|  | **Business card opinion** | Opinion on the business card (content-wise and visually) | “And the QR code, then you go straight to the website I assume? And maybe the idea that for um, new migrants, that immediately just that to, to video or um, so just connected, of just, with their own language is.” |
|  | **Willingness to use business card** | Willingness to give the business card with information about the CSH to other migrant MSM | “Yeah sure, because, point one, I don't really want other people to experience um, the whole worst things I've experienced for example in my life.” |
|  | **Barriers referral CSH** | Barriers for referring other migrant MSM to the CSH by providing the card | “Eh I feel like eh, I would verbally explain like I wouldn’t, I wouldn’t like to be holding this card, I don’t hold any card with me and if I hold, I would lose it, so I will eh, I will just verbally recommend and refer them to the website, so that is what I, I would like same way .. came to me I would do to others.” |
|  | **Facilitators referral CSH** | Facilitators for referring other migrant MSM to the CSH by providing the card | “No, in my flat it's okay. In the flat that's okay, I can give them, no problem. X1: Because it's okay for you to talk openly about it? Y: Yes. X1: Would there be anything that would make it difficult for you to give these cards or nothing? Y: For me no difficult. X1: Nothing. Y: It's good because it's also useful for them. I know there is one also have- One speak Spanish and English but also, he has Dutch boyfriend and he invited him here sometimes also. I can give him this.” |

**Table A.4.2: Codebook professionals**

| Group | Code | Description | Example from data |
| --- | --- | --- | --- |
| Demographics and workplace | **Age (professional)** | Age of the professional | “I just turned 63” |
|  | **Organisation (professional)** | The organisation/department the professional is working | “I work at the GZA.” |
|  | **Position (professional)** | The position of the professional within the organisation | “Sexologist” |
|  | **Field experience (professional)** | How long the professional has been working in the field | “Um, I've been working for, let's see, over eight years with um, um, asylum seekers, status holders, refugees.” |
|  | **STI role organisation (professional)** | What the organisation does to prevent or control STIs *(only asked when applicable)* | “Often they come in with a different complaint and so when you ask through, it does turn out that it's also often um, STI complaints and um, yes, so you do have to try to um, experience that.” |
|  | **STI policy (professional)** | Whether the organisation has an STI policy, and if so, what this policy entails *(only asked when applicable)* | “Actually, I can then prescribe PrEP. I don't have to look at those- Yes, I look at those other STIs at all, but theoretically um, those are not important for me to prescribe PrEP. But you do for the general population, to put it in parentheses, that you think- You don't want to have a, a, a spreader in that group, so you're just spreading all those STIs.” |
|  | **Activities for mMSM by organisation (professional)** | The activities the organisation organizes for mMSM | “So every new resident gets an intake interview upon entry and then whoever that attention officer is, that target group will do that intake interview and um, alert them to the fact that we are there and um, where they can go with their questions and complaints and we will also provide information on paper.” |
|  | **Living situation mMSM (professional)** |  | “They live in the main building and um, that's for vulnerable residents, so people with mental health problems. But they are also vulnerable um, in the shelter, so they also all live together in the main building.” |
|  | **Negative experiences (professional)** | Negative experiences of the professional or the migrants | “Because, of course, they've been through a lot in their home countries, where it's also sometimes punitive or where one is mistreated or eh- And along the way, what do you think what such people there eh, with the appearance- Those, those” |
|  | **Way of contact with mMSM (professional)** | The way the professional comes into contact with mMSM during their profession | “Also occurs, but it is still a smaller group. We do have them living here, those men, and also in Sweikhuizen, but eh- And some then also keep coming with eh, complaints but those are, are really in the minority.” |
|  | **Frequency of contact mMSM (professional)** | How often the professional comes into contact with mMSM per week | “Per month I would say. Once a month that group. So those are then- Yes, um, the majority are men though.” |
|  | **Perceived reach of mMSM (professional)** | Opinion on whether or not mMSM are reached by the professional during their occupation. | “Yes, yes, yes, yes, the general practitioners know where to find us and the GGD knows where to find us too.” |
| HBM / barriers and facilitators to sexual healthcare | **Facilitators experienced by the professional (professional)** | Facilitating factors experienced by the professional during their occupation with mMSM. | “I hear you say, it's in Dutch or in English. We did notice- There was a certain communication policy a year ago, we improved it, and still we kept running into certain things. And when, using all the AI capabilities nowadays, we just had all the policies there translated into Russian, into Arabic, we did make strides in that. Because we have the focus on learning Dutch and English, some who master that, you can get by with that as well. But those people have just spoken a completely different language for thirty, forty years.” |
|  | **Barriers experienced by the professional (professional)** | Barriers experienced by the professional during their occupation with mMSM. | “There is a reason why we as an association do not meet at asylum seekers' centers. That can actually become downright dangerous.” |
|  | **Presumed susceptibility STI mMSM (professional)** | Professional's views on how susceptible mMSM feel to STIs | “Yes, I think so. I think if there is very little knowledge, then you are at risk. X: And do you think they know then than they are at risk? Y: No.” |
|  | **Presumed severity STI mMSM (professional)** | Professional's views on how severity STIs are to mMSM | “Faith plays into some situations I expect. Um, look, I've never had an STD myself, I mean- So I think it's also a very difficult question, because I, I do know people in my um, immediate circle who are HIV-positive um, and those people always, I think, sometimes handle that too easily. Um, but I try to put myself in someone in a vulnerable situation um, you already come here to survive in the hope that you will also have more happiness here in this country. And I think the moment you indeed have in your medical record the label HIV-positive, then, then, then you know that it, that it all gets a little bit trickier- Can be trickier in life, can be. And then you have to have some faith in the medics.” |
|  | **Presumed motivation sexual healthcare mMSM (professional)** | Professional’s opinion on how motivated mMSM are to get sexual healthcare, such as STI tests, (hepatitis B) vaccinations and PrEP/PEP | ” Yeah, yeah, yeah. I just hear people say, I have- Notice now that I can have freer sex. That I, that I do feel a little less anxious then if something happens to that condom.” |
|  | **(Presumed) facilitators sexual healthcare mMSM (professional)** | Professional’s view on what faciliators mMSM might feel to get sexual healthcare, such as STI-tests, (hepatitis B) vaccinations and PrEP/PEP | “Yeah, yeah, yeah. I just hear people say, I have- Notice now that I can have freer sex. That I, that I do feel a little less anxious then if something happens to that condom.” |
|  | **(Presumed) barriers sexual healthcare mMSM (professional)** | Professional's view on whether mMSM might feel barriers (and which ones) to using sexual health services, such as testing for STIs and HIV and vaccination | “Yes, I think they see it as a limitation, that um, other people, for example, realize that they come to us. Or that um, that they realize o, who always goes on Sundays um, there. So that, that can be a, a limitation.” |
| Cues to action and increasing outreach | **Vision of reaching mMSM (professional)** | The professional’s vision on how mMSM can be reached for sexual healthcare services | “Yes, nine times out of ten, someone will come along from the ASC, for example. Then there are already three, then they say, come along too.” |
|  | **Message outreach (professional)** | The message that the professional believes would be appropriate to reach mMSM for sexual health services and how this message should be conveyed | “About eh, the- How it is then arranged from the piece of care. What eh- What options they have and what their own contribution is for that. Which isn't there then. Um- X: That it's free? Y: Yes. Um, and so what they can do preventively as well as reactively.” |
|  | **How to improve services according to the professional (professional)** | The professionals’s vision on how their services for mMSM can be improved (not specifically sexual health related) | “And I think those migrants, really people who have fled, they see any kind of help as, as a plus. But low-threshold and, and, and really focused on nationality, that, to me that would be the dream situation.” |
|  | **Social network for reaching mMSM (professional)** | The professional's opinion on whether or not mMSM's social network can be used to reach other mMSM for sexual health services and why (not)/how. | “Yeah, I think so. I think if you say to the other one of god huh um, who for example has been to a talk and you've then told that and you say of if you know anybody else huh who is struggling with that the same thing, know that we always huh, you can just pass it on, always think that.” |
|  | **Barriers to use social network of mMSM (professional)** | Expected barriers for the mMSM to engage the social network for reaching other mMSM for sexual health services | “With the Arab people, it's a little more difficult anyway. I would say maybe, the Islamic people who are attached to it, also to, to religion, or not? Because it's forbidden as well. Well online, well um, um, through social media, but they also live, they are abroad and um, but not um, in the Netherlands I haven't seen it yet.” |
|  | **Facilitators to use social network of mMSM (professional)** | Expected facilitators for the mMSM to engage the social network for reaching other mMSM for sexual health services | “Yes, that in itself is kind of easy if someone knows the way and tells that too and they also accompany someone here than they come together.” |
|  | **Opinion business card (professional)** | The professional opinion on the business card for mMSM | “I think it's a very clear ticket. I like cards because you have something concrete. X2: Something tangible. Y: Something tangible. You know what I mean? When they save phone number, they save it wrong and can't find it again. Here you have everything under each other and I think, I'm also starting to discover the QR codes a little bit in a general sense- Assume there's some more- Like it says here, more info on these topics.” |
|  | **Expectation distribution of business card (professional)** | The professional's expectation of whether the mMSM will distribute the business card within their network | “A business card like that? I don't know, I think you have to request that per person. I think there are some, who think and then it will end up in the trash, I think that's a waste too. So I think that ehm, yes, I think there's also if they say yes, I know someone who also, because that's also often it is, then it makes sense to give that card along. I think it makes more sense that you put it somewhere, for example, in an um, in a waiting room and you put a couple of them down so they can grab it themselves. But then it's what I also say, of then I wouldn't just do it in English either and would do it in yes, in other languages as well.” |
|  | **Presumed barriers business card (professional)** | The presumed barriers to business card use | “Or if the others eh- Then, they'll hear that, that it's not for them and they could then be referred to us, because then it does get it all done for free, blood draws and urine and, and so, examination.” |
|  | **Presumed facilitators business card (professional)** | The presumed facilitators to business card use | “I think with young people that a QR code like that-because they do everything with their phones-that works really well.” |
|  | **Willingness to give business card (professional)** | The professional's willingness to distribute the business card to mMSM | “Yeah, that would, that really wouldn't matter to me.” |
|  | **Familiarity CSH (professional)** | To what extent the professional is familiar with the work of the Centre for Sexual Health *(only asked when applicable)* | “Yes, this eh- But I did look that up myself once, because indeed I wanted to know what was there, that if someone asks about it, what can I say- So I did know about this, yes.” |
|  | **Cooperation with PHS (professional)** | Whether the professional already collaborates with the Public Health Service's Centre for Sexual Health, and if so how this cooperation is going. | “I do find now that that is um, going very well. Also in the past always.” |
