# Supplementary material for: Perspectives of migrant men who have sex with men and professionals on personal, social and structural barriers and facilitators to sexual healthcare access and outreach strategies: A qualitative study
Source: J Migr Health. 2025 Jun 30;12:100342. doi: 10.1016/j.jmh.2025.100342 (PMC12273491; doi:10.1016/j.jmh.2025.100342)
Supplement: Supplementary file 5 [file mmc5.docx]

**A.5 Barriers and facilitators differentiated by the perceptions**

**Table A.5: Barriers and facilitators differentiated by the perceptions of mMSM and professionals**

| **Level** | **Barriers** | **Facilitators** |
| --- | --- | --- |
| **Individual** mMSM level (reported by mMSM and professional) | **Fear**   - Fear of being stigmatised due to cultural and religious beliefs in the home country - Fear of personal data misuse - Fear of the test result   **Unawareness of services**   - Unaware of existing services for sexual healthcare   **Limited personal resources**   - Limited budget - Lack of transportation   **Competing priorities**   - Other priorities during the asylum process | **Feelings of safety**   - Feelings of safety and comfort in talking about sexual health(services)   **Personal health motivation**   - Desire to know sexual health status |
| **Individual** mMSM level (reported by mMSM) | **Distrust in care**   - Distrust in care (ASC)   **Discomfort**   - Discomfort during STI sample collection   **Unclear sexual orientation**   - Still discovering sexual orientation | **Risk awareness**   - Risk awareness   **Trust in care**   - Trust in the Dutch healthcare system |
| **Individual** mMSM level (reported by professional) | **Distrust in care**   - Distrust in vaccinations   **Testing discomfort**   - Puncture anxiety | **Service awareness**   - Aware of existing services   **Autonomy**   - Independently deciding the course of action concerning testing and treatment or being able to choose which PHS to visit |
| **Interpersonal** mMSM level (reported by mMSM and professional) | **Language difficulties**   - Language barrier - Lack of multilingual staff   **Negative attitude of staff**   - Judgmental or unfriendly nurses (PHS, ASC, GP)   **Staff’s mMSM insensitivity**   - Lack of skills in engaging with different religions - Low motivation in distributing LGBTQ+ materials | **Positive attitude of staff**   - Non-judgmental and friendly nurses (general)   **LGBTQ+ and mMSM sensitivity of staff**   - Migrant and LGBTQ+ sensitivity (ASC, in general) - Professional nurses (PHS)   **Direct contact for appointments**   - Scheduling appointments directly with staff by phone (PHS)   **In-person consultations**   - Face-to-face, in-person consultations   **English proficiency**   - Staff and client interaction in English (ASC, PHS)   **Staff’s and networks support**   - Help from staff, a supervisor or tutor, or network in scheduling appointments - Discussing sexual health(care) with social network   **Trust**   - Consultations with fixed nurse - Confidentiality |
| **Interpersonal** mMSM level (reported by mMSM) | **Lack of person-centred care**   - Complaints not being taken seriously by nurse (ASC) - Lack of attention for sexual orientation (not believed or never asked about) (ASC)   **Non-diverse workforce**   - Lack of multicultural staff (PHS) | **Female nurse**   - Preference for female nurse (PHS) |
| **Interpersonal** mMSM level (reported by professional) | **Distrust**   - Distrust in interpreters | **Male nurse**   - Perceived preference for male staff (ASC) |
| **Interpersonal** professional level (reported by professional) | - | - |
| **Organisational** mMSM level (reported by mMSM and professional) | **Hard-to-find location**   - Hard-to-find office (PHS)   **Far distance to building**   - Far distance to the building (PHS)   **Lack of privacy**   - Lack of privacy in waiting area (PHS, ASC)   **Medical history questionnaire**   - Too many questions (PHS) - Lack of multilingual questionnaire (English and Dutch) (PHS)   **Difficulties scheduling appointment**   - Long waiting for consultation (PHS) - Limited contact hours call centre (PHS)   **Incompleteness of care**   - Incomplete care offer (ASC, GP) - Not specialised in LGBTQ+ care and information provision (GP)   **Elaborate procedures**   - Overly elaborate procedure for STI testing (PHS) | **Manageable distance**   - Manageable distance to building (PHS & ASC)   **Privacy**   - Privacy in the waiting area (PHS & ASC) - STI results by SMS (PHS)   **Complete care offer**   - Comprehensive information provision (PHS, ASC)   **Linguistic services**   - (online) interpreting services (PHS, ASC, in general) |
| **Organisational** mMSM level (reported by mMSM) | **Hard-to-find facility**   - Hard to find the building (PHS) - Lack of route instructions (PHS)   **Complex medical history questionnaire**   - Confrontational questions (PHS) - Not compatible with phone (PHS)   **Lack of (culturally) translated materials**   - Answers options not understandable for newcomers (education levels) (PHS) - Unaware of English version (PHS)   **Difficulties scheduling an appointment**   - Difficulties scheduling an appointment for others (not proficient in Dutch/English) (PHS) - No online booking system (PHS) - No walk-in hours (PHS) - (Too) early morning consultations (PHS)   **Lack of a receptionist**   - No office receptionist for guidance (PHS)   **Incomplete care offer**   - Lack of information on free sexual healthcare for LGBTQ+ (ASC & in general) - Lack of disclosure on coverage of travel expenses (ASC) - Lack of information about service confidentiality (PHS)   **Lack of privacy**   - Not as confidential/private as the PHS (GP)   **Website (un)usability**   - Services do not stand out (PHS) - Difficult to navigate (PHS) - Only in Dutch (PHS) | **Easy-to-find facility**   - Easy to find building (PHS) - Accessible by public transportation (PHS)   **Translated materials**   - Easy and available in English (PHS)   **Ease of scheduling appointment**   - Easy to schedule consultation call centre (PHS) - No long waiting times for consultation (PHS) - Possibility to schedule consultation in advance (PHS)   **Receptionist for directions**   - Office receptionist for guidance (PHS)   **Website usability**   - Clear contact details (PHS) - Public and accessible English information (PHS) |
| **Organisational** mMSM level (reported by professional) | **Incompleteness of care**   - Lack of time per consultation (ASC, GP) - Do not offer free HBV vaccinations (ASC) | **Privacy**   - Privacy in the consultation room (ASC & in general) - Far distance from ASC (PHS) - Safe environment (separate room for consultation) (ASC)   **Easy-to-find facility**   - Fixed location building   **Flexibility in the medical history questionnaire**   - Completion at consultation (PHS) |
| **Organisational** professional level (reported by the professional) | **Low prioritisation**   - Under prioritisation mMSM   **Staff shortages**   - Staff shortage   **Institutional policy**   - Routine STI-testing not provided | - |
| **Community** mMSM level (reported by mMSM and professional) | **Hard to navigate through services**   - New to the healthcare system and its services | **Availability of multiple testing opportunities**   - Multiple locations in one region (PHS & in general) for STI-testing (in general) |
| **Community** mMSM level (reported by mMSM) | - | **Simple to navigate through services**   - Easy to navigate through the healthcare system and its services (PHS) |
| **Community** mMSM level (reported by professional) | - | - |
| **Community** professional level (reported by professional) | **Lack of referral**   - No referral to other services for free HBV vaccination | **Referral**   - Referral between (healthcare) organisations   **Collaboration between organisations**   - Short communication lines between organisations |
| **Societal and policy** mMSM level (reported by mMSM and professional) | **Restrictive financial policies**   - Limited annual (free) STI-checks per individual (without symptoms) (ASC & PHS)   **Strict PrEP policy**   - Changing/difficult PrEP policy (PHS)   **Migrant relocation policies**   - Relocation of migrants   **Lack of legal status**   - Statelessness impedes care reimbursement (general) | **Free care for high-risk groups**   - Free care (PHS, ASC, GP)   **Low costs of PrEP**   - Easy PrEP provision and low costs |
| **Societal and policy** mMSM level (reported by mMSM) | **Geographic service limitation**   - Uncertain which PHS to visit geographically   **GP gatekeeping**   - Can't go to a specialist without going to GP first (general)   **High diagnostics costs**   - High costs for diagnostics at the GP | **Open societal norm**   - Societal norm of openness about sexual health and sexuality   **Societal STI-awareness**   - Perceived high STI awareness in the country   **Legal protection against discrimination**   - Discrimination ban in constitution: Dutch law prohibits discrimination based on sexual orientation |
| **Societal and policy** mMSM level (reported by professional) | - | - |
| **Societal and policy** professional level (reported by professional) | **Restrictive financial policies**   - Limited budget based on region | **Political support**   - Politically endorsed funding |
